# Dr. John B. Hamilton

**Published:** 1899-02

**Authors:** 


Dr. John B. Hamilton, of Chicago, died at his residence at Elgin, Ill., Saturday, December 24, 1898, aged 51 years. The immediate cause of his death was hemorrhage, due to perforation of the intestine. This perforation communicated with a large abscess outside the bowel, and the disease had continued for something less than a month.

Dr. Hamilton was one of the most conspicuous figures in American medicine. He entered the military service during the Civil War at the age of 17. Three years after the close of the war he took his medical degree at Rush Medical College, Chicago. Soon afterward he entered the U. S. Army as assistant surgeon. In 1876 he was transferred to the U. S. Marine Hospital service, in which he rapidly rose by promotion to be Surgeon-General. He molded the corps into a distinctly efficient body, and in 1888 made a personal contest with the yellow-fever epidemic in the South. In 1892 he resigned the office of Surgeon-General and removed to Chicago, retaining the office of surgeon in the corps, and became professor of surgery in his alma mater. In 1893 he became editor of the Journal of the American Medical Association, which place he held at his death. In 1896 he was appointed Superintendent of the Northern Illinois Hospital for the Insane at Elgin, in which service he died.

Dr. Hamilton was authority upon questions of public health, was a surgeon by instinct and training, an executive officer of first ability, a scholar, a gentleman and a most charming man professionally, socially and officially. This brief sketch of his life does sadly imperfect justice to one of the most brilliant and picturesque careers that American medicine has witnessed. He died young, but he had accomplished more than falls to most men whose spans of life run the allotted period. He will be missed everywhere that medical men meet.

